# Syringomyelia in the Course of Resection of Foramen Magnum Pathology: A Case Report with an Intriguing Therapeutic Solution and Systematic Review

**DOI:** 10.3390/brainsci16010014

**Published:** 2025-12-22

**Authors:** Rafal Rajski, Waldemar Kolodziej, Tomasz Sobolewski, Krzysztof Kandziora, Tomasz Klepinowski, Anna Latka, Beata Labuz-Roszak, Dariusz Latka, Kajetan Latka

**Affiliations:** 1 Doctoral School, University of Opole, 45-060 Opole, Poland; 2 Department of Neurosurgery, University Hospital, Institute of Medical Sciences, University of Opole, 45-040 Opole, Poland; 3 Helimed—Imaging Diagnostics, St. Hedwig’s Regional Specialist Hospital, 45-221 Opole, Poland; 4 Department of Neurosurgery, Pomeranian Medical University, 70-204 Szczecin, Poland; 5 Department of Neurology, St. Hedwig’s Regional Specialist Hospital, Institute of Medical Sciences, University of Opole, 45-040 Opole, Poland; 6 Department of Neurosurgery, St. Hedwig’s Regional Specialist Hospital, 45-221 Opole, Poland

**Keywords:** syringomyelia, tumor resection complication, foramen magnum tumor

## Abstract

Introduction: Syringomyelia is a rare disease of the spinal cord, and postoperative syringomyelia is an extremely rare complication. There are very few scientific reports on this subject in the literature, which is why we decided to review them and report our case of a patient after resection of a meningioma of the foramen magnum; we diagnosed syringomyelia in the course of postoperative complications. Methods: PubMed MEDLINE, Scopus, and Web of Science were searched. The PRISMA checklist was followed for a systematic structure. We paid special attention to publications describing syringomyelia only as a complication of invasive procedures and not as a pathology co-occurring with other diseases. In addition, our studies were based exclusively on the adult population. Results: As a result of the literature analysis, we selected 5 papers that met the established criteria, which are case studies describing the 5 patients. In each of them, syringomyelia was diagnosed after a procedure on the central nervous system (or spine). In three papers, it was decided to treat the syringomyelia that developed in this way surgically, and in the remaining two, conservative treatment and adjustment of the already inserted ventriculoperitoneal shunt were performed. The case of a patient described by us after resection of a meningioma of the foramen magnum required surgical treatment of syringomyelia after two years due to significant progression of the syrinx and the occurrence of troublesome clinical symptoms. In all cases described (including ours), significant clinical improvement was ultimately achieved, allowing patients to return to their daily activities. Conclusions: Iatrogenic syringomyelia is an extremely rare complication that is poorly described in the literature. The large heterogeneity and small number of reported cases make it difficult to draw clear conclusions. Further studies and analyses describing this topic are necessary, and will allow for a better understanding of the mechanism and standardized treatment. However, based on our work, we suggest that interventions on the ventricular system in particular may increase the probability of postoperative syringomyelia.

## 1. Introduction

Syringomyelia, characterized by the presence of pathological cavities within the spinal cord, poses significant neurological challenges and consequences [1]. Although the etiology of syringomyelia is diverse, it is frequently associated with Chiari malformation, spinal cord tumors, trauma, and can also emerge as a complication of surgical interventions [1]. The pathophysiology of syringomyelia is not fully understood, and several theories have been developed to solve this medical mystery. A detailed description and critical analysis of these theories is beyond the scope of this publication, so we have focused on a brief introduction to each of them by listing them in Table 1.

The symptoms of postoperative syringomyelia do not seem to differ from those of other etiologies and mainly include dissociated sensory loss, muscle weakness that is often asymmetrical, as well as gait disturbances and sphincter disorders [7]. Historically, syringomyelia has been described as a consequence of tumor pathologies in the foramen magnum [8].

The diagnosis and management of syringomyelia remain a challenge for neurosurgeons and neurologists due to the complexity of the mechanisms leading to its development and its varied clinical manifestations. Effective management of this condition requires accurate diagnosis, monitoring of disease progression, and individualized therapy, which may include both conservative treatment and surgical interventions [7].

This article presents the case of a patient who developed syringomyelia after resection of a large foramen magnum meningioma, along with a comprehensive review of the literature on similar cases. This case highlights the complex interaction between the anatomical structures of the foramen magnum and the dynamics of the cerebrospinal fluid (CSF). It highlights the need for innovative therapeutic approaches in the treatment of this complication. The aim of this article is to present an interesting and extremely rare complication of foramen magnum meningioma resection, for which the epidemiological frequency has not been estimated yet. It details the surgical intervention, postoperative management, and ultimately the successful therapeutic outcome. It also provides a detailed analysis of the literature on similar cases, with particular emphasis on the procedure performed, the course of symptoms, and the implemented management. This case highlights the complexity of this complication and highlights various treatment strategies.

## 2. Methods

The literature review was conducted based on the PRISMA 2020 checklist. After the authors’ consensus, we did not register the review in PROSPERO. In accordance with the PRISMA 2020 guidelines [8], a review of medical literature was performed in May–July 2024, including case reports, systematic reviews, etc., describing syringomyelia as a complication of invasive procedures (surgeries, injections) within the central nervous system. The review was performed in the PubMed MEDLINE, Web of Science, and Scopus scientific databases using the following keywords: syringomyelia; syringohydromyelia; complication; postoperative; surgery; postsurgery; iatrogenic; and de novo with (AND) combination. The results were presented in diagrams and tables in other sections of this study. Due to significant heterogeneity, the results were presented mainly in descriptive form.

In order to examine the issue in greater detail, strict inclusion and exclusion criteria were applied. The inclusion criteria were as follows: publication written in English; age over 18 years; what is essential for our study—syringomyelia as a complication of an invasive procedure; and full-text version of the paper. Exclusion criteria were as follows: age under 18 years; co-occurrence of Arnold-Chiari syndrome; syringomyelia not as a complication but co-occurring with a tumor; and paper with only an abstract available online. There were no restrictions on year of publication, funding sources, country of origin, size of the research group, or type of publication.

**Figure 1:**
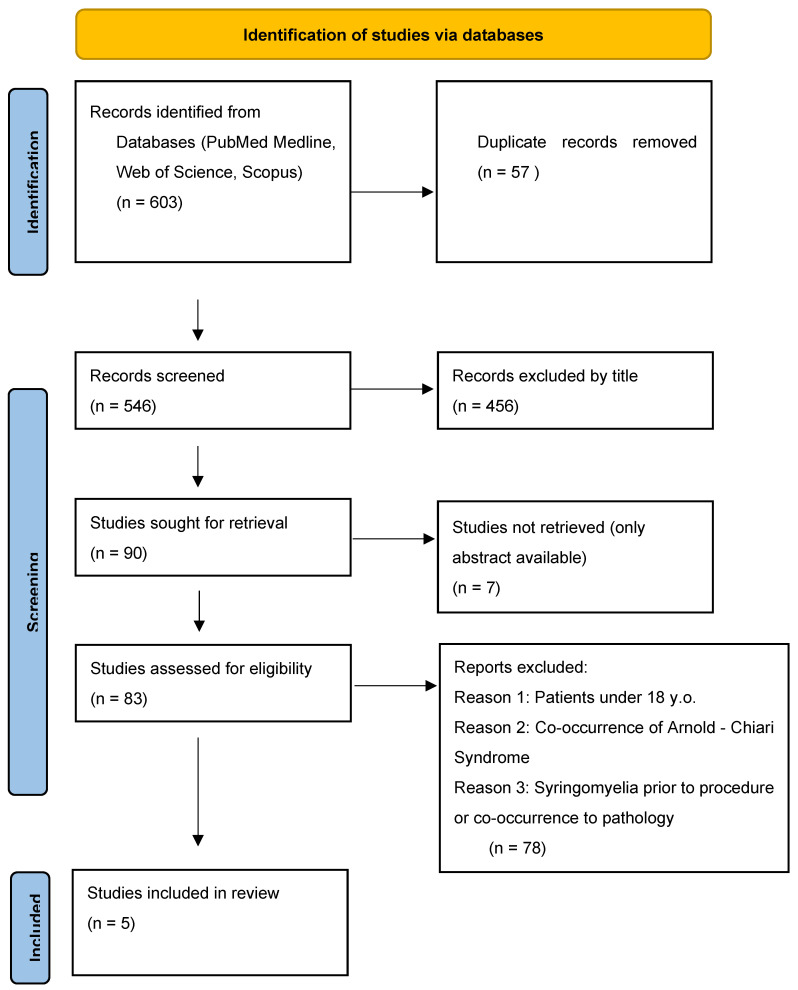
PRISMA 2020 flow diagram showing the selection proces of publications for the analysis.

Literature review and selection of papers were performed by two authors. Two authors simultaneously conducted a literature review, initial publication selection, abstract evaluation, and exclusion of papers that did not meet the established criteria. Particular attention in the text analysis was paid to the occurrence of syringomyelia as a complication of a given procedure, and not as a co-occurring condition, as well as rejection of papers describing patients with Arnold-Chiari syndrome, which is the most common and best-described factor of syringomyelia, and its occurrence would make it difficult to determine other risk factors. After selecting the final number of publications, both authors separately assessed the content of the papers. The subsequent stages of selection are presented in the PRISMA2020 flow diagram (Figure 1) [8].

In the first stage of database review, 603 articles were identified based on keywords, from which 57 duplicates had to be excluded. In the next step, the selected publications were assessed by the authors based on their titles, specifically whether they included phrases such as “complication,” “syringomyelia,” “brain tumor resection,” “invasive procedure,” etc. Exclusive terms included “pediatric,” “Arnold-Chiari Syndrome,” “co-occurrence of syringomyelia,” etc.

Then, 90 publications were qualified for full-text evaluation, of which only an abstract was available for 7 publications. During the initial full-text review, special attention was paid to exclusion criteria. Furthermore, the type of study (original articles, systematic reviews, and meta-analyses) was assessed for the quality of statistical analysis, a well-conducted assessment of risk of bias, description of the procedures performed, and a thorough assessment of syringomyelia and its relationship to the procedure. Again, it was important to exclude publications involving patients with Arnold-Chiari syndrome or those under 18 years of age. An additional advantage was the accurate consideration of demographic data such as age structure and gender. As a result of this review, five publications were selected, which were case studies. At the detailed evaluation stage, the authors worked separately to assess the quality of the publications using the CHECKLIST FOR CASE REPORTS questionnaire, which is a Critical Appraisal tool for use in JBI Systematic Reviews [9]. In the questionnaire, the case study was analyzed based on seven control questions assessing the description of essential elements of this type of publication. The response options were “YES,” which we awarded 1 point, “UNCLEAR” for 0.5 points, and “NO” for 0 points. We determined that for a case study to qualify, the reviewer must receive a minimum of 5 points, representing the arithmetic mean of the responses of the two authors. Rounding was performed in accordance with generally accepted mathematical principles to the nearest 0.5 points. Finally, 5 selected studies met our criteria and were subjected to further analysis (Table 2). Due to the inability to use advanced statistical evaluation tools, we focused primarily on descriptive presentation of the results.

In the case report section, we focus on presenting a patient who initially underwent surgical resection of a foramen magnum meningioma diagnosed using a head MRI. A detailed description of the diagnosis and treatment is provided later in this publication.

## 3. Results

After evaluating the results and applying rigorous exclusion criteria, 5 case studies were obtained that met the basic assumptions of the work, in particular, the occurrence of syringomyelia as a complication of the invasive procedure, and not its co-occurrence. The information subjected to analysis was collected in Table 3 and Table 4. Due to the high heterogeneity of the cases, we decided to include information from medical history, which was considered important from the point of view of our study.

The presented analysis compares 5 patients aged 26 to 57 years. Only one paper [12] included a patient with no history of intervention on the ventricular system of the brain. The remaining four patients [8,10,11,13] underwent ventricular drainage of the brain at some point in their lives, with two of them due to hydrocephalus [9,10], one due to idiopathic intracranial hypertension [13], and one as a result of complications after resection of a vestibular schwannoma tumor [8]. The time of diagnosis of syringomyelia from the time of the procedure to confirmation in MRI ranged from the period immediately after the procedure to 4 years. In one paper, the authors decided to treat syringomyelia conservatively [12], while in another, the procedure consisted of regulating the VP shunt [12]. In three studies [8,10,11], surgical intervention was performed via the suboccipital approach with decompression of the posterior temporal fossa and removal of the obstruction to the flow in the ventricular system. All papers describe the cure of syringomyelia indicating improvement in clinical symptoms, and MRI image details specific to each publication are described in Table 3 and Table 4.

### Case Report

**Figure 2:**
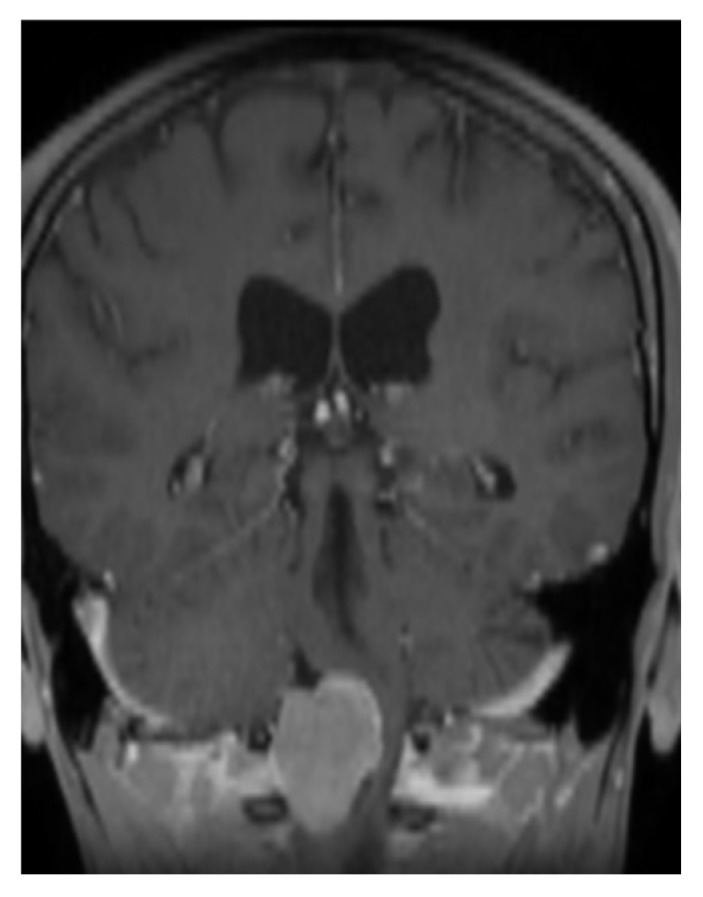
Single coronal scan of a right-sided foramen magnum meningioma with mild brainstem modeling. MRI T1 + C.

**Figure 3:**
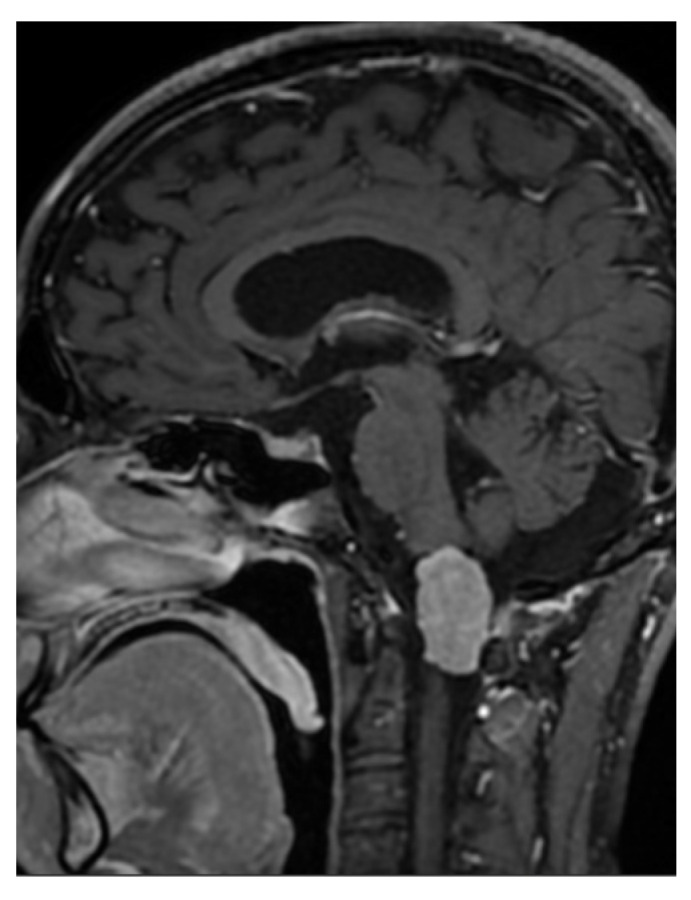
Single sagittal scan of a right-sided foramen magnum meningioma with mild brainstem modeling, MRI T1 + C.

**Figure 4:**
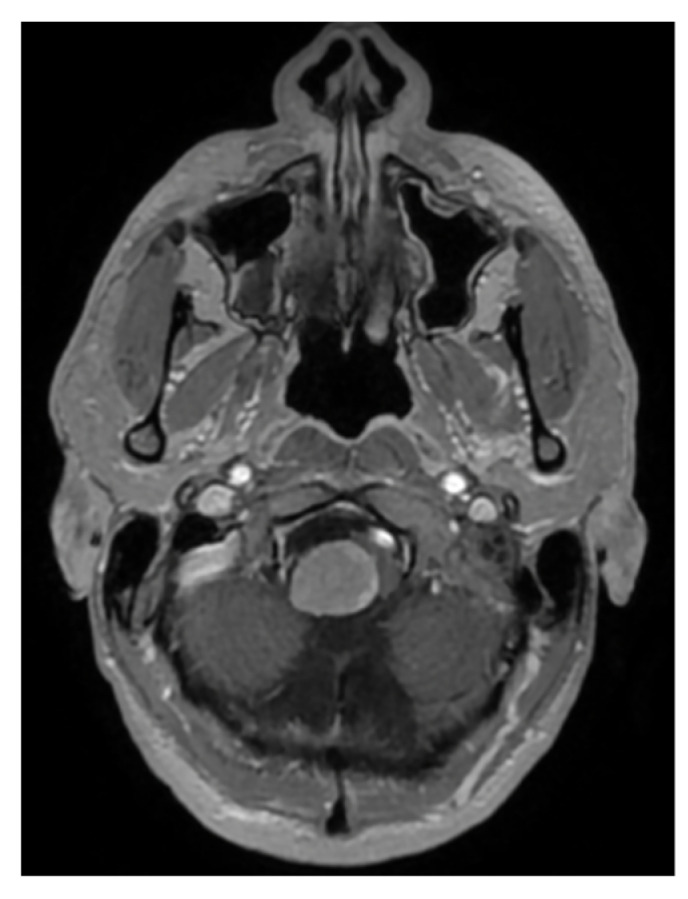
Single cross-sectional scan of a right-sided foramen magnum meningioma with mild brainstem modeling. MRI T1 + C.

A 33-year-old patient sought a neurosurgical consultation due to persistent, stubborn neck pains. MRI examination revealed a large tumor located in the right lateral part of the foramen magnum, with the morphology of a meningioma (Figure 2, Figure 3 and Figure 4).

**Figure 5:**
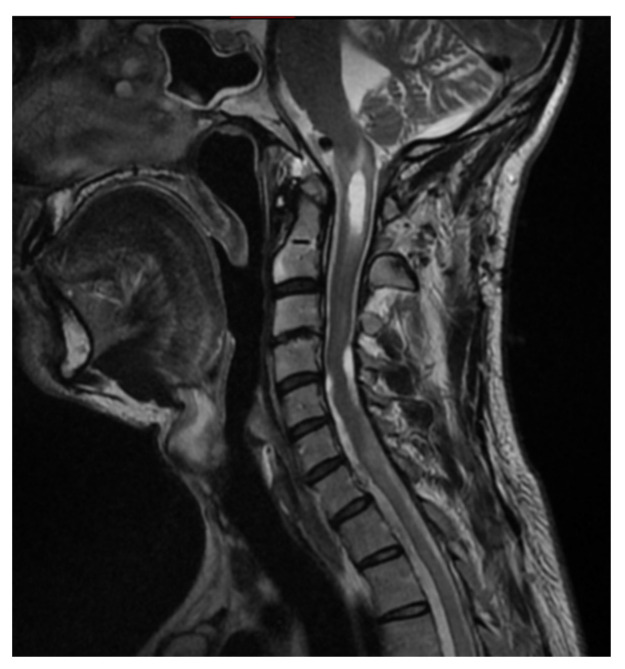
Patient 4 weeks after tumor resection with enlarged central canal of the cervical spinal cord. Single Sagittal Scan MRI T2.

The patient was qualified for surgical treatment and underwent a procedure through a right far lateral approach. The course of the operation was successful. On the first day post-operation, the patient was mobilized without neurological deficits. CT control identified a hematoma located in the fourth ventricle but without enlargement of the ventricular system. On the second day, there was a deterioration in contact, and hiccup occurred. CT control revealed obstructive hydrocephalus, leading to the decision to implant a ventricular drain. The patient’s condition improved in terms of consciousness post-drainage implantation, though hiccup persisted. After 2 weeks, CT control showed absorption of the blood, and the drainage was removed after a closure test. A few days after functioning well, the patient’s state of consciousness deteriorated again. In the subsequent control CT, hydrocephalus was again observed, leading to the decision to implant a ventriculoperitoneal (VP) shunt. Post-shunt implantation, the patient presented periodic improvement in contact, enabling mobilization and verticalization with periods of significant consciousness decline. This condition persisted for two weeks, during which the patient was hospitalized in neurosurgery. After 2 weeks, the patient suddenly returned to a completely normal state of consciousness, and all neurological deficits ceased. Before discharge, a control MRI of the head and spine was performed, which showed enlargement of the spinal cord’s central canal (Figure 5). However, given the very good clinical condition of the patient, he was discharged home.

**Figure 6:**
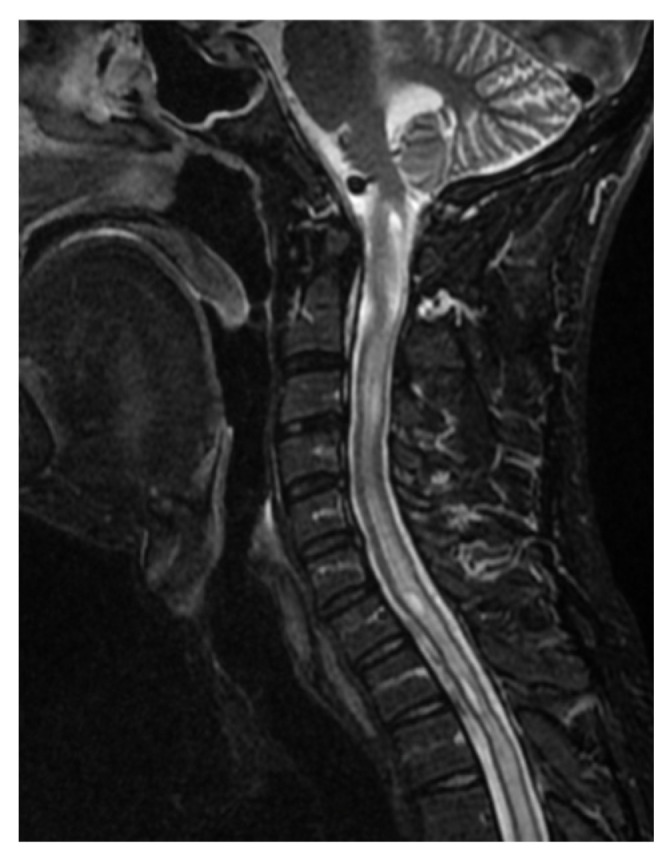
Significant progression of syringomyelia involving the entire spinal cord. Cervical spine. Single Sagittal Scan. MRI T2.

The patient functioned normally for 2 years, returning to professional activity and was continuously monitored, and subsequent MRI examinations showed gradual progression of syringomyelic cavities within the spinal cord. Nevertheless, given his good overall condition, he opted for further observation. After 2 years, he experienced disturbances characterized by sensory disturbances in the left half of the body and balance disorders. He was admitted for neurological diagnostics, where Lyme disease was diagnosed, and after antibiotic treatment, the disturbances slightly decreased but did not completely withdraw. In control MR, advanced syringomyelia of the entire spinal cord was observed (Figure 6).

**Figure 7:**
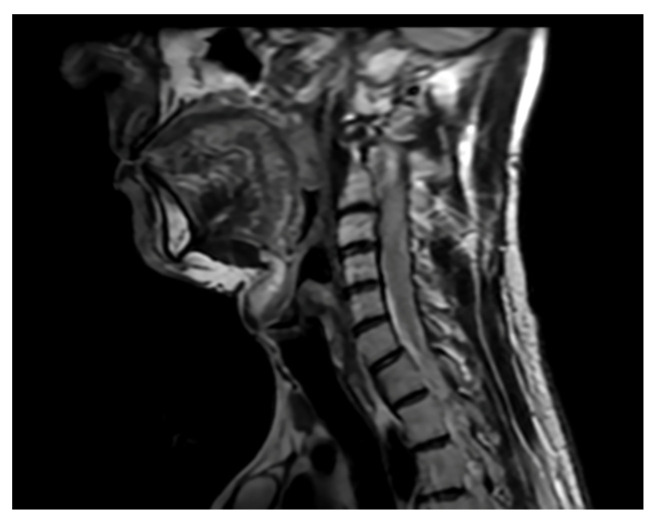
Single sagittal scan of the cervical spine. Follow-up MRI 2 months postoperatively. MRI T2.

After a thorough analysis of the case, the decision was made to proceed with surgical treatment. An exploration of the fourth ventricle was decided upon. Intraoperatively, a very thickened arachnoid membrane covering the cerebellum was observed. After opening the fourth ventricle, a duraplasty was performed to enlarge the space over the cerebellar tonsils. After the procedure, the patient reported improvement in balance and slightly less paresthesia. Control MR showed syringomyelic cavities of the same morphology as before the procedure. Additionally, postoperative changes in the bed of hemorrhagic nature were observed; however, these did not cause significant edema. Two months later, in MR control, significant remission of syringomyelic cavities was noted (Figure 7). The patient returned to normal functioning.

## 4. Discussion

Syringomyelia is a rare disease of the spinal cord and sometimes also of the brainstem, characterized by local dilatation of the central canal of the spinal cord, usually occurring in the cervical spine, but with a tendency to extend to other segments. An even rarer variant of syringomyelia is the formation of cavities outside the central canal [1]. The epidemiology of this disease has been taken up in several publications, according to which its incidence does not exceed 9/100,000 [14,15].

The most important symptoms suggesting syringomyelia are as follows: dissociated sensory loss, muscle weakness often asymmetric, related to the section where the syrinx has formed. In addition, patients complain of gait disturbances and sphincter disorders. In the case of syringomyelia at the level of the brainstem (also called syringobulbia), additional symptoms may include hoarseness, dysphagia, cough with difficulty swallowing, visual disturbances, hearing disturbances, etc. [1,7,16,17,18]. According to the studies, up to 23% of syringomyelia may be asymptomatic and accidentally detected [19]. The gold standard in diagnostics is MRI, which allows for achieving up to 100% sensitivity [1,20]. Other imaging tests used to diagnose syringomyelia are as follows: Dynamic MRI [20]; Cardiac-Gated Cine MRI Flow Study [21]; and Myelography with High-Resolution Computed Tomography Scan [22].

The etiology of syringomyelia is very diverse, but the most important predisposing factor is Arnold-Chiari syndrome, in which the structures of the hindbrain are displaced into the spinal canal. According to the literature, it is responsible for about 2/3 of diagnosed syringomyelia [15]. Other causes include meningitis [23], arachnoiditis [24], trauma [25], advanced spinal stenosis [26], and central nervous system tumors [27], including those located in the area of the foramen magnum (e.g., medulloblastoma, meningioma) [28]. The patient described in our case with meningioma of the foramen magnum was also at risk of syringomyelia, and indeed, a case study and even systematic reviews of the coexistence of both pathologies have been described in the literature [27]. Interestingly, there are extremely few studies that describe the occurrence of syringomyelia as a complication of resection of such a tumor [8], which is why we decided to explore this topic.

The very small number of scientific reports on iatrogenic syringomyelia and the need to apply strict exclusion criteria meant that the final number of analyzed papers was quite low and was associated with some heterogeneity. This is particularly related to the poor epidemiology of this pathology, the varied clinical course, and the difficulties in treatment and long-term patient follow-up. This last reason, in particular, makes it difficult to produce high-quality scientific reports, let alone conduct systematic reviews that would allow for statistical assessment. Ultimately, after thorough selection and evaluation, only five publications were eligible for analysis. Because the selected works were case studies, we were unable to conduct extensive statistical analysis; instead, we focused on a detailed description and comparison of the reported cases. Especially interesting is the fact that in 4 of the 5 publications [4,5,7,8], the patients had undergone some procedure on the ventricular system (mainly VP shunt) in their medical history. Comparatively, our patient also required VP shunt placement (less than 3 weeks after meningioma resection), and after another two weeks, a control MRI scan showed central canal dilatation. Surgery within the foramen magnum, due to the presence of significant cerebrospinal fluid pathways, is primarily associated with the risk of developing hydrocephalus, which seems logical. However, the subsequent dilation of the central canal, especially after a properly placed VPS, is surprising. This raises the question of whether the syringomyelia we described is a complication of VPS resection or implantation. The rapid and extensive enlargement of the syringomyelic cavity is particularly intriguing. It is difficult to find clear evidence for the cause of this progression, but we speculate that it may be related to changes in cerebrospinal fluid flow following tumor resection, which chronically disrupts its flow, as well as VPS implantation, which is an additional factor disrupting physiological circulation within the ventricular system. Another potential factor in the development of syringomyelia may be arachnoid adhesions, which we observed during reoperation in our patient. These adhesions were likely related to a hematoma within the fourth ventricle that occurred after the primary resection. We thoroughly removed the adhesions, opening the fourth ventricle and performing duraplasty, which widened the space for the cerebellar tonsils. It is difficult to definitively determine the extent to which these adhesions may have impaired CSF flow or how the duraplasty improved its flow pathway. However, their significant contribution to this pathology is evidenced by the fact that after removal of the pathologically altered arachnoid and increasing the space at the craniocervical junction, the patient’s condition rapidly improved and the syringomyelic cavities gradually regressed. Unfortunately, we are unable to determine what had a greater impact on the development of syringomyelia, but we postulate that in our patient’s case several factors combined, primarily arachnoid adhesions and procedures on the ventricular system (VPS implantation). In the cited publications, we see a significant discrepancy in the time from surgery to diagnosis of syringomyelia, which makes it difficult to determine certain trends. In each case, MRI scan allowed for easy diagnosis and, due to the bothersome clinical symptoms, surgical treatment (3 of the 5 publications) [4,5,8]. Particularly interesting and at the same time difficult to understand is the case of Khan S. et al. [12], where the patient did not undergo any major neurosurgical operation (including on the ventricular system) and the fact that syringomyelia developed immediately after the procedure. The procedure also differs from the other works, in which the authors decided on conservative treatment, which was only described as steroid boluses (short-term before transfer to another center) and oxygen therapy. The remaining procedures were not described.

Surgical treatment of syringomyelia is a very broad topic that requires a lot of experience [1]. The assessment of intracranial compliance is important from the point of view of long-term results. Therefore, patients who had a ventriculoperitoneal shunt placed before decompression treatment achieve regression of syringomyelia in 80% [29]. In the case of patients without VP shunt, resistant to previous conservative or surgical treatment and in four-chamber hydrocephalus, additional stent placement and/or dilatation of the section between the fourth ventricle and the subarachnoid space of the spine may be necessary [30].

The accepted indications for surgery are as follows: symptomatic Chiari Malformation—(CM1) with a syringomyelic cavity; asymptomatic CM1 with a holocord syrinx with Vaquero index > 0.5 or coexisting syringobulbia; symptomatic isolated syringomyelia; posttraumatic syringomyelia with movement disorders [1,2]. Surgical treatment is largely dependent on the initial cause of syringomyelia, but its main assumption is based on the osteo-meningeal decompression of the posterior cranial fossa and the removal of obstruction within the foramen magnum and the foramen of Magendie [31].

There are many variations in the procedure, such as the co-occurrence of CM-1 when additional craniocervical decompression is necessary with opening the dura and arachnoid with removal of the adhesions, as a result of such a procedure an artificially enlarged foramen magnum is created, allowing free flow of CSF [32]. In the case when the patient has additional hydrocephalus, it is necessary to insert a shunt or endoscopic ventriculostomy [33]. If necessary, additional interventions are allowed, such as dilatation of the fourth ventricle [34], detethering of the spinal cord [35], stabilization of the spine [36], etc. The lack of effectiveness of the above-mentioned methods may suggest the need for syrinx shunting, but the effectiveness of such a procedure is very controversial, with a high risk of complications [37]. Properly treated syringomyelia allows for a high success rate in both clinical and radiological assessment; in the case described by us and in the cited publications [4,5,6,7,8], it was possible to achieve almost complete remission of symptoms, allowing patients to return to their daily activities.

Our study is based on the adult patient population (>18 y.o.), because if we included the pediatric population in the analysis due to its high heterogeneity and significant anatomical and physiological differences, we would not have obtained the most similar data for comparison. In our opinion, we should cite the work of Kurzbuch AR. et al. [38], in which the authors, despite focusing exclusively on iatrogenic syringomyelia after skull surgery in children, collected rich material for conducting a systematic review with a high level of evidence. They found that the most common causes of such a complication are, in order as follows: CM-1 decompression, VP shunt, and, less frequently, resection of intracranial tumors.

Based on epidemiological data and estimated numbers of neurosurgical procedures in the skull or even the spine, we believe that the number of reports on postoperative syringomyelia seems to be surprisingly small. The lack of publication of reports in this area or any other rare complications leads to a serious underestimation of the scale of complications, difficulties in understanding the pathomechanism, and problems with establishing a standardized therapeutic procedure based on meta-analyses and systematic reviews, and not only on incidental cases described as case studies. We hope that the number of publications on syringomyelia will increase and in the future, we will be able to perform a more extensive analysis and share our experiences.

## 5. Conclusions

Syringomyelia and iatrogenic syringomyelia are rare and still little-known pathologies in medicine. Our case of a patient with syringomyelia after resection of a foramen magnum meningioma is considered a medical casuistic approach, as evidenced by the small number of reports in the largest medical databases on this or a similar topic, which mainly consists of case studies. We suggest that perhaps CSF flow disorders and VP shunt implantation may affect the subsequent formation of syringomyelia, but confirmation of such a thesis would require description and analysis of a significantly larger number of cases. Despite the lack of strong scientific evidence and highly standardized therapeutic procedures in the treatment of syringomyelia, all patients described in this work achieved remission of cavities, with the withdrawal of clinical symptoms enabling return to daily activity. In the future, it is necessary to repeat the literature review on this topic, taking into account a larger number of clinical cases.

## Figures and Tables

**Table 1:** Summary of theories attempting to explain the mechanism of syringomyelia development [2,3,4,5,6].

Author	Theory
Gardner and Angel [2]	The so-called “hydrodynamic theory”, in which the hindbrain’s hernias obstruct the CSF flow, which then diverts into the central canal and causes gradual widening of the canal by pulsation resulting from the heart’s work (“water-hammer” effect).
Wiliams [3]	During Valsalva maneuvers, gradients exceeding 100 mm Hg across the foramen magnum cause suctional force that draws ventricular CSF into the syrinx cavity. The entered fluid impacts the walls, causing their dilation.
Ball and Dayan [4]	Increased spinal CSF pressure during coughing or other activity leads to increased pressure gradients, and with a foramen magnum obstruction, this causes CSF to flow through the Virchow-Robin spaces along the spinal cord surface.
Oldfield et al. [5]	The CSF pressure wave generated during systole causes a caudal spinal cord displacement which forces the fluid to enter the syrinx cavities and subarachnoid spaces.
Koyanagi, and Houkinkoya [6]	Non-physiological rapid movement of CSF through the central canal promotes the creation of a low-pressure zone within the canal, leading the extracellular fluid to concentrate and form a syrinx.

**Table 2:** Quality assessment 0–7 (points) of selected publications conducted using CHECKLIST FOR CASE REPORTS from Critical Appraisal tool for use in JBI Systematic Reviews [9].

Publication	Year/Ref.	Author I	Author II	Average
Honorato D.	2004 [10]	6	6	6
Peraud A.	2009 [11]	7	6.5	7
Khan S	2010 [12]	5	6	5.5
Kersey J.	2017 [13]	7	7	7
Egu C.B.	2021 [8]	6.5	6	6.5

**Table 3:** General characteristics of the analyzed publications and described patients, including basic statistical information, a brief background, and medical procedures [8,10,11,12,13].

Author	Year	Patient History	Procedure Performed	Time from Surgery to the Occurrence of Syringomyelia
**Honorato D. [10]**	2004	33 y.o. female with hydrocephalus treated with ventriculoperitoneal (VP) shunt. After 3 months, surgical removal of cysticercoid cyst in the left temporal region was conducted with additional pharmacological treatment. After a few months, neuroinfection of unknown etiology occurred. In March 2000, the patient experienced frequent dizziness, nausea, and occasional vomiting. An MRI examination showed diffuse arachnoiditis and a cyst in the IV ventricle which was treated with a craniectomy of the posterior fossa with the IV ventricle cyst removal. Over the next few months, neuroinfection occurred again and a VP shunt replacement was conducted.	Posterior fossa craniectomy with IV ventricle cyst removal/VP shunt replacement.	14 months (VP shunt replacement)/16 months (posterior fossa craniectomy).
**Peraud A. et al. [11]**	2009	33 y.o. female. Hydrocephalus at the age of 16 was treated with a VP shunt. In the meantime, the patient experienced 3 pregnancies.	Natural childbirth (3rd).	1 week.
**Khan S. et al. [12]**	2010	52 y.o. female suffering from chronic neck pain.	Cervical epidural injection (CEI) under fluoroscopy.	Immediately after the procedure.
**Kersey J. [13]**	2017	37 y.o. female with idiopathic intracranial hypertension; headache; and visual obscurations.	VP shunt.	Following months.
**Egu C.B. [8]**	2021	26 y.o. female diagnosed with right-sided cystic vestibular schwannoma. Absent right-sided hearing at presentation and patient reported that this had deteriorated over the preceding 7 years.	Right-sided retrosigmoid craniec tomy and complete excision.	4 years.

**Table 4:** Comparison of symptoms, MRI results, and treatment of patients in selected publications, along with the follow-up described in the publication [8,10,11,12,13].

Author	Symptoms Related to the Occurrence of Syringomyelia	MRI Findings	Treatment	Follow-Up
**Honorato D. [10]**	Asymptomatic, then 5 months after syringomyelia diagnosis, progressive paraparesis (with massive syrinx progression in control MRI).	Dilatation of the IV ventricle due to obstruction of the inferior aqueductorifice and the lateral and median foramens of the IV ventricle. Cervical syringohydromyelia and suffusion in the medullar parenchyma.	Posterior fossa exposure, widening the previous craniectomy, and communicating the hydrosyringomyelia with the subarachnoid space.	In the control MRI, reestablishment of normal CSF dynamics in the posterior fossa with reduction of the cervical syringohydromyelic cavity.
**Peraud A. et al. [11]**	Rapidly progressive spastic tetraparesis more pronounced on the legs. Motor strength grade 2/5 on the lower and 4/5 on the upper extremities with brisk reflexes.	Extensive syringomyelia involving the entire spinal cord, generalized ventricular enlargement, as well as cystic-appearing alterations in the fourth ventricle.	Operation on 5th day after the delivery. A suboccipital trepanation with resection of the fourth ventricular cyst and an insertion of a new VP shunt with a programmable valve.	MRI—reduced flow in the spinal syrinx as a veritable sign for a significant drainage; 5th day post-op motor deficits recovery. Two years after surgery, patient regained the ability to walk without support. No remaining sensory deficits are present. Urine bladder has regained some function.
**Khan S. et al. [12]**	Inability to move right arm and leg; numbness of the right side of the body below the neck; and urinary retention.	C1 to Th4 Syrinx, without mass effect.	A high dose of steroids were administered; after patient was transferred to another hospital, steroids were discontinued, and conservative treatment was applied.	At discharge, resolution of urinary retention. No more details mentioned.
**Kersey J. [13]**	Mild mid-thoracic pain with no neurological deficits.	cervicothoracic syrinx	Regulation the VP shunt pressure after the delivery.	At one year postpartum there had been no progression of symptoms. The valve was set at 2.0.
**Egu C.B. [8]**	After the surgery, four admissions over the next 4 months with intermittent headaches, nausea, and craniectomy site swelling. CT revealed a pseudomeningocoele with hydrocephalus. Therapeutic lumbar punctures and drainage during each admission. In the meantime, the patient experienced several infections and worsening of symptoms. Progressive worsening of headaches, bilateral hands paresthesia, and vomiting.	Enlarged fourth ventricle with outflow obstruction, plugging of the foramen magnum, and an extensive new upper cervical cord syrinx.	Posterior fossa and upper cervical decompression and drainage of the syrinx were performed on account of the enlarged fourth ventricle with outflow obstruction, plugging of the foramen magnum and an extensive new upper cervical cord syrinx occurring 4 years following the initial right- sided retrosigmoid craniectomy and complete excision of the incidental vestibular schwannoma.	Symptoms improved and she was discharged with her VP shunt functioning well. For 5 years, there was a complete resolution of symptoms.

## Data Availability

The original contributions presented in this study are included in the article. Further inquiries can be directed to the corresponding author.
